# A Homozygous Deep Intronic SNX14 Variant Activates Pseudo-Exon Inclusion in a Patient with SCAR20

**DOI:** 10.3390/genes17040378

**Published:** 2026-03-26

**Authors:** Doriana Misceo, Petter Strømme, Arvind Y. M. Sundaram, Pål Marius Bjørnstad, Mari Elen Strand, Maninder Singh Chawla, Eirik Frengen

**Affiliations:** 1 Department of Medical Genetics, Oslo University Hospital and University of Oslo, 0450 Oslo, Norway; arvind.sundaram@medisin.uio.no (A.Y.M.S.); p.m.bjornstad@medisin.uio.no (P.M.B.); m.e.strand@medisin.uio.no (M.E.S.); eirik.frengen@medisin.uio.no (E.F.); 2 Division of Pediatrics and Adolescent Medicine, Oslo University Hospital, 0450 Oslo and Faculty of Medicine, University of Oslo, 0450 Oslo, Norway; petter.stromme@medisin.uio.no; 3 Department of Neuroradiology, Oslo University Hospital, 0450 Oslo, Norway; mancha@ous-hf.no

**Keywords:** cerebellar ataxia, deep intronic variant, FRASER 2.0, OUTRIDER, RNA-sequencing, SCAR20, *SNX14*

## Abstract

**Background**: The contribution of intronic variants to the etiology of Mendelian diseases is still underrecognized, impacting the diagnostic yield. Whole genome sequencing (WGS) detects intronic variants, but besides canonical splice-sites, intronic variants are frequently excluded from the interpretation step or are classified as variants of uncertain significance (VUS). In fact, assessing their clinical significance often requires validation via RNA-sequencing (RNA-seq) or in vitro studies. **Methods**: We studied a 31-year-old patient with spinocerebellar ataxia who lacked a molecular diagnosis after WGS analysis. We applied the Detection of RNA Outliers Pipeline (DROP) to analyze RNA-seq data from patient fibroblasts. DROP integrates OUTRIDER and FRASER 2.0 algorithms designed to identify aberrant gene expression and splicing, respectively. **Results**: DROP identified differential expression and aberrant splicing of *SNX14*. Retrospective WGS data analysis revealed a homozygous NM_153816.6(SNX14): c.867+288A>G deep intronic variant, which caused pseudo-exon activation and reduced transcript levels. Biallelic loss-of-function variants in *SNX14* cause autosomal recessive spinocerebellar ataxia type 20 (SCAR20; OMIM 616354), consistent with the clinical presentation of this case. **Conclusions**: We identify a deep intronic *SNX14* variant as the genetic basis of SCAR20. We demonstrate the utility of RNA-seq to increase the diagnostic yield by identifying and resolving the pathogenicity of deep intronic variants. Defining aberrant splicing events is therapeutically relevant, as these mechanisms are targets for antisense oligonucleotide (ASO) based interventions.

## 1. Introduction

Despite the technological advances in DNA sequencing, approximately 50% of Mendelian disorders remain genetically unresolved after whole exome and whole genome sequencing (WES and WGS) [1]. One challenge to improving this statistic is the annotation and interpretation of intronic variants not categorized as canonical splice-sites. Examples of such DNA changes are deep intronic variants that are located more than 100 bp away from the closest exon-intron boundary [2]. These variants can disrupt normal splicing, and alternative splice isoforms can create frameshifts and/or premature stop codons that are pathogenic [3]. While intronic variants are robustly identified by WGS, their functional impact is difficult to predict with in silico tools, which limits their diagnostic yield as many remain classified as variants of unknown significance (VUS) [2,4]. RNA-sequencing (RNA-seq) has emerged as a powerful complementary analysis enabling the detection of aberrant gene expression and aberrant splicing that pinpoint genes harboring pathogenic changes. Using this approach, RNA-seq guides targeted retrospective examination of WGS data to identify clinically relevant pathogenic variants [5,6]. Detection of RNA Outliers Pipeline (DROP) is an integrative workflow that detects differential expressions and aberrant splicing from RNA-seq data [7]. DROP integrates two statistical algorithms to prioritize disease-relevant changes: OUTRIDER, for the detection of expression outliers, and FRASER 2.0, for the detection of splicing outliers [8,9]. Transcriptomics was used to identify the genetic etiology of a 31-year-old individual with a neurodegenerative disease characterized by spinocerebellar ataxia and profound intellectual disability, the genetic basis of which was still unresolved after WGS analysis. DROP analysis of RNA-seq data from fibroblasts derived from the affected individual identified differential expression and aberrant splicing of *SNX14*. Retrospective analysis of WGS data, based on transcriptomic findings, revealed a homozygous deep intronic variant NM_153816.6(SNX14): c.867+288A>G, GRCh37/hg19: chr6:g.86,257,731T>C, p.?, responsible for pseudo-exon activation and reduced transcript levels. Biallelic pathogenic variants in Sorting nexin 14 *(SNX14*) cause SCAR20 (Spinocerebellar Ataxia, Autosomal Recessive 20; OMIM 616354), a condition overlapping with the clinical features of the affected individual. This study documents the pathogenicity of a deep intronic *SNX14* variant and highlights the diagnostic value of RNA-seq analysis as an important tool to identify and assess pathogenic deep intronic variants that would otherwise remain VUS.

## 2. Materials and Methods

### 2.1. Whole Genome Sequencing (WGS) and Data Analysis

Genomic DNA was extracted from the peripheral blood of the patient. Sample preparation for WGS was done using TruSeq^TM^ PCR-free library preparation kit (Illumina, San Diego, CA, USA). WGS was performed using the Illumina HiSeq X instrument (Illumina, San Diego, CA, USA) with 150 bp paired-end reads. The data were analyzed using the nf-core/raredisease pipeline (version 2.2.0) [10]. The reads were aligned to the GRCh38 human reference genome with BWA-MEM2 (version 2.2.1) [11]. Duplicate reads were marked using Picard MarkDuplicates (version 3.3.0) (https://broadinstitute.github.io/picard/, accessed on 1 June 2025). Variant calling of each sample was performed with DeepVariant (version 1.6.1, gVCF mode) [12]. Joint genotyping of the trio was then conducted using GLnexus (version 1.4.1) [13]. Functional annotation was performed by the Ensembl Variant Effect Predictor (VEP, version 110) [14], and variant deleteriousness scores were computed by the Combined Annotation Dependent Depletion tool (CADD, version 1.6) [15]. The tools vcfanno (version 0.3.5) [16] and BCFtools (version 1.20) [17] were used to annotate variants with population allele frequencies from gnomAD (version 4.1) [18], clinical significance from ClinVar (downloaded on 19 September 2024) [19] and splice junction prediction scores from SpliceAI [20]. The final variant calling file (VCF) was analyzed using the FILTUS program [21]. We discarded variants with allelic frequency > 0.01 in gnomAD (version 4.1.0). We also discarded variants with a CADD PHRED score < 10. We focused on single-nucleotide variants (SNVs), small insertion/deletion (Indels) variants causing missense, nonsense, frameshifts, or affecting splice-sites. We used the AuTEX: autozygosity function in FILTUS to identify regions of autozygosity in the WGS data of the proband. We searched for homozygosity regions longer than 1 centi Morgan (cM) and containing more than 15 variants.

### 2.2. RNA-Seq and Data Analysis

Fibroblasts from skin biopsies of the proband and a cohort of 40 additional samples were cultivated in DMEM, high glucose (Gibco, Waltham, MA, USA) with 10% FBS, 100 U/mL penicillin, and 100 μg/mL streptomycin at 37 °C in 5% CO_2_. The cohort consisted of skin biopsies from children with a genetic disease of known or unknown etiology and from healthy adults. The individuals, 47% female and 53% male, were of varied ancestry.

RNA was extracted from cultured fibroblasts using the Ambion PARIS™ system (Thermo Fisher, Waltham, MA, USA). Samples were prepared for RNA-seq with the Illumina Strand-specific TruSeq mRNA-seq library prep (Illumina Inc., San Diego, CA, USA). The libraries were indexed, pooled, and sequenced on an Illumina Novaseq X (Illumina Inc.) with 150 bp paired-end reads.

Reads were aligned to the ENSEMBL reference GRCh37 release 87 (Homo_sapiens.GRCh37.dna.primary_assembly.fa and Homo_sapiens.GRCh37.87.gtf) with STAR aligner (version 2.7.11b) [22] in two-pass mode using the Tomte pipeline (version 3.0.0) (https://github.com/genomic-medicine-sweden/tomte, accessed on 1 June 2025), which orchestrates RNA-seq processing and integrates DROP [7] for aberrant expression and splicing analyses. Within this framework, statistical algorithm OUTRIDER and FRASER 2.0 were used for the detection of aberrant expression and aberrant splicing, respectively [8,9]. In OUTRIDER, we used *p-adjusted* values ≤ 0.05 as the cut-off for calling significant expression outliers [8]. In FRASER 2.0, split reads spanning the exon-exon junction and non-split reads spanning the splice sites were counted for splicing event calling [9]. The Intron Jaccard Index is computed using split and non-split reads for capturing several types of aberrant splicing. Significant aberrant splicing events were defined with *p-adjusted* values ≤ 0.1 [9]. The analysis cohort included 40 additional fibroblast samples to model background variation. Aberrant events were visually inspected using Integrative Genomics Viewer IGV (IGV version 2.19.1) [23].

## 3. Results

### 3.1. Clinical Presentation

**Figure 1:**
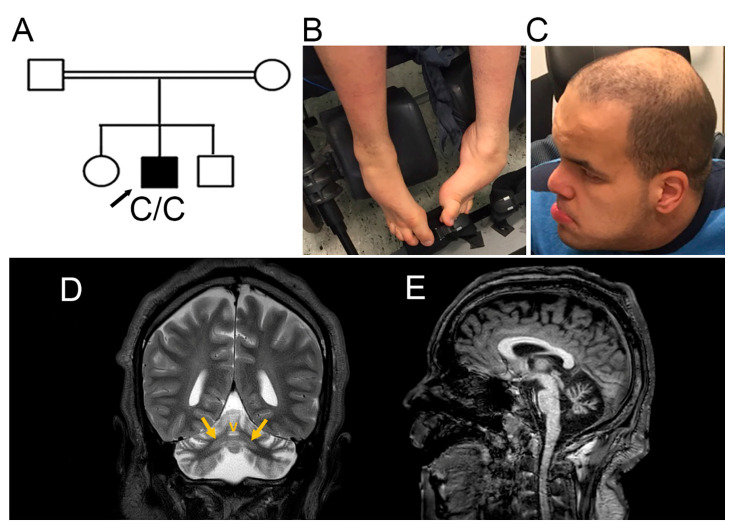
(**A**) Pedigree of the patient’s family. The patient was homozygous for a single-nucleotide variant in *SNX14*: Chr6(GRCh37): g.86,257,731T>C. (**B**,**C**) Photos of the patient at 22 years. (**B**) Lower extremities showing dystonic posture and talipes equinovarus. (**C**) The patient presented a prominent forehead, deep-set eyes, bushy eyebrows, and thick lips. Alopecia was also evident. (**D**,**E**) Brain MRI of the patient at 21 years. (**D**) Coronal T2-weighted image shows both cerebellar hemispheres and vermis (V) atrophy with bilateral symmetric dentate nucleus hyperintensity (arrows). (**E**) Sagittal T1-weighted image shows pronounced cerebellar vermis atrophy.

The patient was a male born in 1995 to a consanguineous union of Moroccan Berber descent that also had two unaffected children (Figure 1A). Pregnancy and delivery at term were uneventful. Signs of developmental delay were evident early on, beginning with poor head control, delayed visual tracking and eye contact, and the absence of consonant babbling. He sat with support at 1 year, and without support at 7–8 years, though this acquired skill was later lost. Walking with independence was not achieved.

At 2 years, he experienced a seizure and was treated with antiepileptic medication for four years. After a decade, epilepsy recurred and has been managed with antiepileptic medication to date. Hearing impairment was suspected at age 2. Brainstem auditory evoked response tested at 3 years showed thresholds > 80 dB, consistent with neurogenic deafness.

From 8 years of age, he developed dystonic posturing of the feet, later confirmed as equinovarus deformities. He also developed scoliosis that was managed with orthotic bracing. Alopecia began around 16 years of age.

When last examined at Oslo University Hospital at 22 years, the proband was wheelchair-bound with markedly reduced mobility, particularly in his lower limbs. No purposeful or spontaneous movements were observed. Muscle tone was increased with dystonic posturing, most prominent in the lower extremities, and equinovarus positioning (Figure 1B) was more pronounced when awake. Deep tendon reflexes were difficult to elicit, but bilateral plantar responses were extensor with toe fanning. No definite cranial nerve deficits were noted, although visual tracking was limited. Sensory function appeared preserved.

Cognitive development was profoundly impaired. He did not have expressive verbal language and produced only limited vocalizations. He exhibited stereotypic facial and hand movements (Supplementary file Video S1). Dysmorphic features included coarse facial traits, a prominent forehead, deep-set eyes, thick bushy eyebrows, thick lips, and a broad chin (Figure 1C). He was macrocephalic, with a head circumference of 62 cm (2.5 cm > 97th centile at 21 years). The proband was unable to attend a recent follow-up visit due to wheelchair transportation issues. According to his mother and legal guardian, his clinical condition has been stable since the last visit, aside from the recurrence of epilepsy.

Brain MRI performed at 11 and 21 years revealed atrophy of both cerebellar hemispheres and vermis (Figure 1D,E) with bilateral symmetric dentate nucleus hyperintensity (Figure 1D). There were no abnormal signal changes in the basal ganglia. MR spectroscopy (MRS) from the right cerebellar hemisphere showed elevated glutamate and myoinositol with preserved choline peaks, creatine, *N*-acetyl aspartate (NAA) peaks, and normal lactate. These findings were interpreted as nonspecific but compatible with a degenerative disease [24]. Diagnostic 105k aCGH (Agilent Technologies, Santa Clara, CA, USA) was normal.

### 3.2. Variant Identification by DROP on RNA-Seq Data

**Figure 2:**
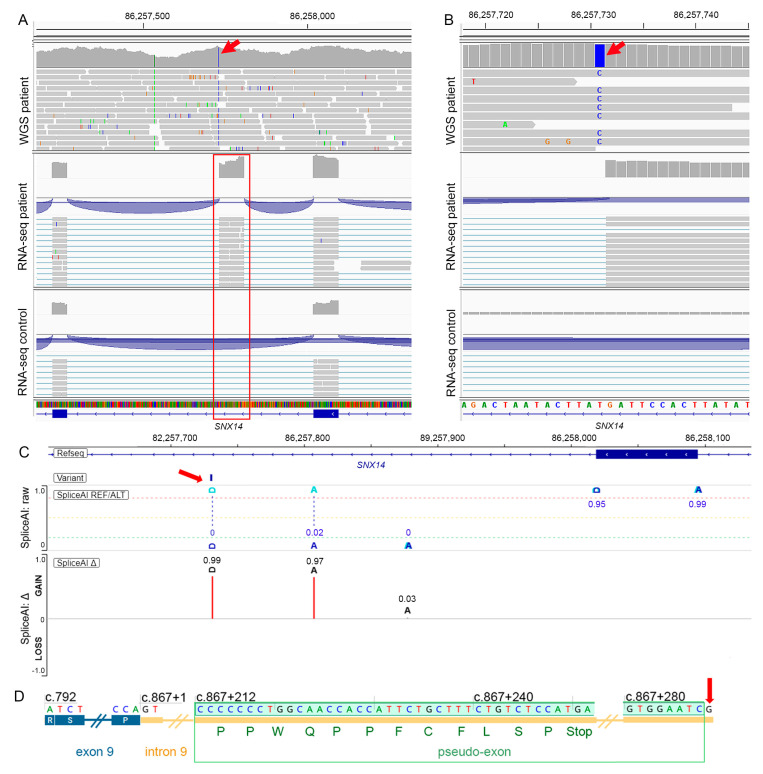
(**A**) IGV screenshot of RNA-seq data showing the *SNX14* homozygous variant chr6:86,257,731T>C (red arrow) in the patient (**top** panel) and pseudo-exon inclusion event at chr6:86,257,732–86,257,807 (red box). A representative control sample showing no transcription of this intronic region (**bottom** panel). (**B**) Zoom-in of the intronic region surrounding the homozygous variant. (**Top** panel): WGS of the patient. (**Middle** panel): RNA-seq of the patient showing pseudo-exon inclusion. (**Bottom** panel): RNA-seq of a representative control sample. The red arrow indicates the variant. (**C**) Visualization of the SpliceAI prediction of the *SNX14*: chr6:86,257,731T>C variant (red arrow). The SpliceAI algorithm predicted the intronic variant to create a splice donor site (Δ = 0.99) at chr6:86,257,732 bp, and an acceptor site (Δ = 0.97) at chr6:86,257,807 bp. (**D**) Schematic of the pseudo-exon activation caused by the NM_153816.6(SNX14):c.867+288A>G (red arrow). The variant leads to the inclusion of a pseudo-exon (green) within intron 9. Translation of the pseudo-exon introduces a premature stop codon after 12 amino acids (green letters), likely triggering NMD. Exons are shown as blue boxes, introns as yellow lines, and the pseudo-exon is highlighted in green. All genomic positions refer to GRCh37 and are shown in bp.

RNA-seq analysis using DROP revealed differential expressions and aberrant splicing of *SNX14* (Table S1A,B). In OUTRIDER, *SNX14* expression was found reduced (*p* adjusted value 5.35 × 10^−4^, log_2_ fold change ≈ −2.17). In FRASER 2.0, we detected aberrant inclusion of *SNX14* intron 9 (chr6:86,257,269–86,258,018 bp) (*p* adjusted value 1.82 × 10^−8^) consistent with a pseudo-exon activation mechanism. Review of *SNX14* transcripts in IGV demonstrated transcription of a pseudo-exon at chr6:86,257,732–86,257,807 bp, which was not observed in any other samples from the cohort (Figure 2A, Supplementary Figure S1). RNA-seq data from the proband showed no *SNX14* transcripts with normal splicing. STAR was used for read alignment and junction detection. While other aligners were not evaluated, the depth and consistency of the junction-spanning reads support the validity of the pseudo-exon inclusion event.

### 3.3. Retrospective WGS Data Analysis

The initial WGS analysis did not identify any putative pathogenic variants. Diagnostic yield for this analysis was hampered by the limited tools to annotate deep intronic variants to permit prioritization for pathogenicity. Following detection of the pseudo-exon by DROP, we reanalyzed WGS data at the *SNX14* locus and identified the following variant in homozygosity: NM_153816.6(SNX14):c.867+288A>G, GRCh37/hg19: chr6: g.86,257,731T>C, p.? aberrant splicing event (Figure 2B). The SpliceAI algorithm predicted the creation of a splice donor site (Δ = 0.99) at chr6:86,257,732 bp, and an acceptor site (Δ = 0.97) at chr6:86,257,807 bp (Figure 2C), matching the pseudo-exon coordinates revealed by the RNA-seq data. Inclusion of this pseudo-exon produced a frameshift that created a premature stop codon: NP_722523.1 (SNX14): p. (Asp290Profs*12) (Figure 2D). The premature stop codon likely triggered nonsense-mediated decay (NMD), consistent with reduced *SNX14* expression detected by OUTRIDER. The variant was not reported in gnomAD and had a CADD PHRED score of 12.99 in GRCh37-v1.6 and 24 in GRCh37-v1.7. The variant resided in a region of homozygosity of 9.2 Mb (chr6:81,933,223–91,131,421 bp). Structural variant calling on the WGS data did not identify any structural variants affecting *SNX14*. The parents were not available to confirm the inheritance pattern; however, with the consanguinity and the 9.2 Mb region of homozygosity encompassing the *SNX14* locus, the most parsimonious explanation is that both parents were heterozygous carriers.

Using ACMG/AMP criteria [25,26], the NM_153816.6(SNX14):c.867+288A>G variant was classified as likely pathogenic: based on PVS1_Moderate (RNA-seq demonstrated pseudo-exon inclusion resulting in a frameshift and premature stop codon in a gene where loss of function is an established disease mechanism), PM2 (absence from gnomAD), and PP4 (highly specific clinical phenotype).

To our knowledge, alopecia is not a typical feature of SCAR20 and may be caused by a variant in another gene, which we were unable to identify.

## 4. Discussion

We report a homozygous deep intronic *SNX14* variant in an individual presenting with features of SCAR20. RNA-seq analysis of fibroblasts revealed reduced *SNX14* transcript levels and aberrant splicing. Retrospective WGS analysis of the *SNX14* locus revealed a noncoding variant in intron 9 that introduced a pseudo-exon and produced a loss-of-function *SNX14* allele, in line with the genetic mechanism of SCAR20.

The clinical impact of deep intronic variants is poorly understood and is therefore possibly underreported as a cause of Mendelian disorders [2,4]. To date, more than 40 individuals with SCAR20 have been described, with the majority harboring *SNX14* nonsense, frameshift, or canonical splice-site variants that function according to a loss-of-function mechanism [27,28,29,30,31,32,33,34,35,36,37,38]. In contrast, only one deep intronic variant (NM_153816.6:c.462-589A>G) has been described in two affected sisters [39]. We expand the *SNX14* allelic spectrum beyond coding and canonical splice-site variants, highlighting the pathogenic relevance of non-coding variants in SCAR20.

The neurological functions of *SNX14* are conserved in mammals. Constitutive loss of *Snx14* in mice causes embryonic lethality, while conditional *Snx14* deletion in neurons and glia and results in Purkinje cell degeneration and severe motor deficits [40]. At cellular and molecular level, conditional loss of *Snx14* disrupts microtubule organization and mitochondrial transport through destabilization of the spastin enzyme, leading to impaired axonal integrity and cerebellar ataxia [40]. SNX14 also localizes to endoplasmic reticulum–lipid droplet contact sites and plays a key role in lipid homeostasis, autophagy, and organelle crosstalk, and its loss results in accumulation of lipid droplets, enlarged lysosomes, and impaired autophagosome clearance [27,36,41]. While these SNX14 pathogenic mechanisms have yet to be confirmed in human-derived affected cell types, similar pathogenesis can account for the early onset global developmental delay, macrocephaly, coarse facial features and cerebellar atrophy with ataxia and spasticity detected in the affected individual we describe here, and that also falls within the phenotypic spectrum of congenital disorders of autophagy [42,43].

Beyond their diagnostic importance, the identification of cryptic splice events has high therapeutic value. Pseudo-exon activation represents a pathogenic allele with a targetable mechanism. ASOs are being successfully used to mask aberrant splice sites, restoring normal splicing. Proof of concept for this strategy has already been demonstrated in several genetic disorders, including Leber congenital amaurosis type 10 (OMIM 611755)caused by the deep intronic *CEP290* variant NM_025114.4:c.2991+1655A>G, for which ASO-based therapies have advanced to clinical trials [44,45].

## 5. Conclusions

This study expands the spectrum of *SNX14* pathogenic variants by describing a novel pseudo-exon activating deep intronic variants. The variant was identified through RNA-seq analysis, highlighting a limitation of WGS-based diagnostics alone. The study demonstrates how RNA-seq can provide a functional readout to interpret intronic variants that are challenging to annotate into clinically relevant findings.

## Data Availability

The data will be available upon request. Distribution of sensitive data may be subject to restrictions.

## Supplementary Materials

The following supporting information can be downloaded at https://www.mdpi.com/article/10.3390/genes17040378/s1. Video S1. The patient at the age of 22 years, wheelchair-bound and showing repetitive head and hand movements. Table S1. DROP analysis results showing *SNX14* as gene expression outlier (panel A) and splicing outlier (panel B) in the patient compared to the cohort. Figure S1: IGV visualization of patient RNA-seq data (three biological replicates) compared with nine controls. The pseudo-exon inclusion in *SNX14* is evident in all three patient samples, while no inclusion is detected in any of the nine control samples

## Abbreviations

The following abbreviations are used in this manuscript:

ACMG/AMP	American College of Medical Genetics and Genomics/ Association for Molecular Pathology
CADD	Combined Annotation Dependent Depletion
DROP	Detection of RNA Outliers Pipeline
FDR	False discovery rate
IGV	Integrative Genomics Viewer
Indels	Small insertion/deletion
NAA	N-acetyl aspartate
NMD	Nonsense-mediated decay
RNA-seq	RNA-sequencing
SNVs	Single Nucleotide Variants
SNX14	Sorting nexin 14
SCAR20	Spinocerebellar ataxia, autosomal recessive 20
VUS	Variants of uncertain significance
WES	Whole exome sequencing
WGS	Whole genome sequencing
