# Supplementary figures and images for: A Homozygous Deep Intronic SNX14 Variant Activates Pseudo-Exon Inclusion in a Patient with SCAR20
Source: Genes (Basel). 2026 Mar 26;17(4):378. doi: 10.3390/genes17040378 (PMC13116672; doi:10.3390/genes17040378)

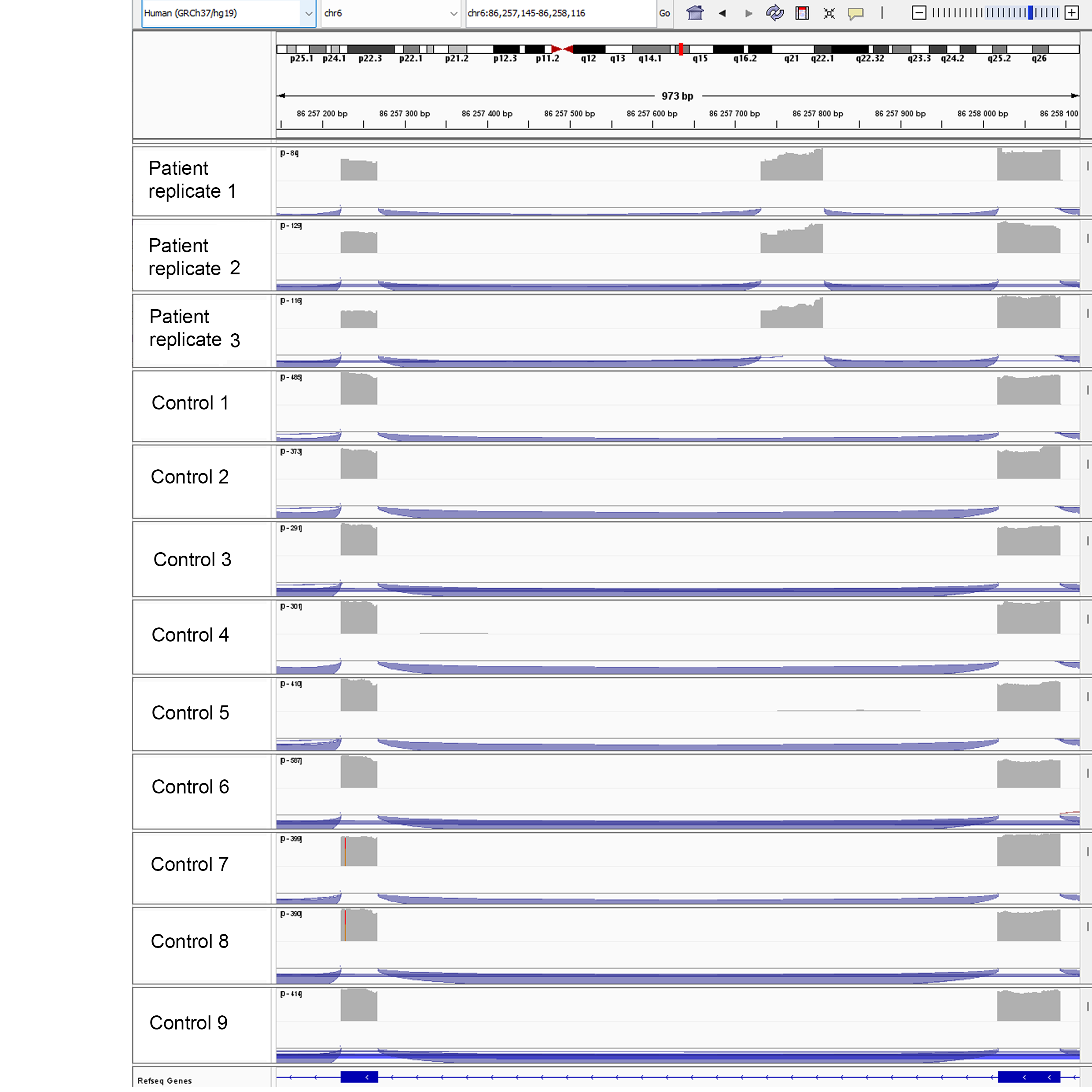

Supplement: Supplementary file 1 [file genes-17-00378-s001.zip › Supplementary files/SNX14 Supplementary Figure 1.tif]
